# Tinnitus after Simultaneous and Sequential Bilateral Cochlear Implantation

**DOI:** 10.3389/fsurg.2017.00065

**Published:** 2017-11-13

**Authors:** Geerte G. J. Ramakers, Véronique J. C. Kraaijenga, Yvette E. Smulders, Alice van Zon, Inge Stegeman, Robert J. Stokroos, Rolien H. Free, Johan H. M. Frijns, Wendy J. Huinck, Gijsbert A. Van Zanten, Wilko Grolman

**Affiliations:** ^1^Department of Otorhinolaryngology, Head and Neck Surgery, University Medical Center Utrecht, Utrecht University, Utrecht, Netherlands; ^2^Brain Center Rudolf Magnus, University Medical Center Utrecht, Utrecht, Netherlands; ^3^Department of Otorhinolaryngology, Head and Neck Surgery, Maastricht University Medical Center, Maastricht, Netherlands; ^4^Department of Otorhinolaryngology, University Medical Center Groningen, Groningen, Netherlands; ^5^Graduate School of Medical Sciences, Research School of Behavioural and Cognitive Neurosciences, University Medical Center Groningen, Groningen, Netherlands; ^6^Department of Otorhinolaryngology, Head and Neck Surgery, Leiden University Medical Center, Leiden, Netherlands; ^7^Leiden Institute for Brain and Cognition, Leiden University Medical Center, Leiden, Netherlands; ^8^Department of Otorhinolaryngology, Head and Neck Surgery, Radboud University Medical Center, Nijmegen, Netherlands; ^9^Donders Institute for Brain, Cognition and Behaviour, Radboud University Medical Center, Nijmegen, Netherlands

**Keywords:** tinnitus, cochlear implantation, bilateral cochlear implantation, simultaneous, sequential, Tinnitus Handicap Inventory, Tinnitus Questionnaire

## Abstract

**Importance:**

There is an ongoing global discussion on whether or not bilateral cochlear implantation should be standard care for bilateral deafness. Contrary to unilateral cochlear implantation, however, little is known about the effect of bilateral cochlear implantation on tinnitus.

**Objective:**

To investigate tinnitus outcomes 1 year after bilateral cochlear implantation. Secondarily, to compare tinnitus outcomes between simultaneous and sequential bilateral cochlear implantation and to investigate long-term follow-up (3 years).

**Study design:**

This study is a secondary analysis as part of a multicenter randomized controlled trial.

**Methods:**

Thirty-eight postlingually deafened adults were included in the original trial, in which the presence of tinnitus was not an inclusion criterion. All participants received cochlear implants (CIs) because of profound hearing loss. Nineteen participants received bilateral CIs simultaneously and 19 participants received bilateral CIs sequentially with an inter-implant interval of 2 years. The prevalence and severity of tinnitus before and after simultaneous and sequential bilateral cochlear implantation were measured preoperatively and each year after implantation with the Tinnitus Handicap Inventory (THI) and Tinnitus Questionnaire (TQ).

**Results:**

The prevalence of preoperative tinnitus was 42% (16/38). One year after bilateral implantation, there was a median difference of −8 (inter-quartile range (IQR): −28 to 4) in THI score and −9 (IQR: −17 to −9) in TQ score in the participants with preoperative tinnitus. Induction of tinnitus occurred in five participants, all in the simultaneous group, in the year after bilateral implantation. Although the preoperative and also the postoperative median THI and TQ scores were higher in the simultaneous group, the median difference scores were equal in both groups. In the simultaneous group, tinnitus scores fluctuated in the 3 years after implantation. In the sequential group, four patients had an additional benefit of the second CI: a total suppression of tinnitus compared with their unilateral situation.

**Conclusion:**

While bilateral cochlear implantation can have a positive effect on preoperative tinnitus complaints, the induction of (temporary or permanent) tinnitus was also reported.

**Clinical Trial Registration:**

Dutch Trial Register NTR1722.

## Introduction

Tinnitus is a common symptom in patients with profound sensorineural hearing loss (SNHL). Currently, standard clinical care for adult patients with bilateral profound SNHL in the Netherlands is unilateral cochlear implantation. Prevalence rates of preoperative tinnitus in cochlear implant (CI) patients range from 66 to 86% (1). Although cochlear implantation is indicated for the hearing loss, a suppression of tinnitus is often reported as a beneficial side effect (2).

Several hypotheses exist about the etiology of tinnitus. It is thought that maladaptive plasticity in the auditory nervous system can result in tinnitus (3). One hypothesis is that lack of peripheral auditory input leads to an overactivity of the central auditory system, which manifests as the perception of tinnitus (3, 4). Following this hypothesis, restoring the peripheral auditory input (with a CI) could lead to a decrease of tinnitus perception.

A recent systematic review showed a decrease in mean tinnitus burden after cochlear implantation in all 10 included studies (2). On individual level, the majority of patients with preoperative tinnitus benefited from cochlear implantation (suppression rates between 8 and 45%, decrease rates between 25 and 72%); however, some patients experienced an increase in tinnitus (0–25%). In addition, newly induced tinnitus after cochlear implantation is reported (rates varying between 0 and 20%) (2, 5, 6). Cochlear implantation as a treatment for invalidating tinnitus in patients with unilateral hearing loss is still part of debate in the literature; however, short-term as well as long-term studies show promising results (7–10).

There is an ongoing global discussion on whether or not bilateral cochlear implantation should be standard care for bilateral deafness. Contrary to unilateral cochlear implantation, however, only a few studies reported on the effect of bilateral cochlear implantation on tinnitus (11–13). Our study group previously investigated tinnitus burden 1 year after unilateral compared with simultaneous bilateral cochlear implantation (13). No statistically significant differences were found between the two study groups. In a study by Summerfield et al. (11), 24 unilateral CI users received a second CI with a median inter-implant interval of 2.7 years (inter-quartile range (IQR): 1.7 years). Remarkably, the mean tinnitus scores in the whole study group increased after receiving the second CI. In 7 of the 16 patients who reported tinnitus preoperatively, the tinnitus worsened after receiving the second CI. Also, in four of the eight patients without preoperative tinnitus, newly induced tinnitus occurred after receiving the second CI. In a retrospective study by Olze et al. (12), 40 sequentially bilaterally implanted patients, with a mean inter-implant interval of 3.6 years (range: 0.35–15.9 years) were evaluated. The tinnitus scores of the 28 patients with preoperative tinnitus decreased after the first CI and even further after the second CI. None of the 12 patients without preoperative tinnitus developed tinnitus postoperatively.

As the results of the above-mentioned studies are contradictive, no firm conclusions can be drawn about the additional effect of a second CI on tinnitus. Therefore, the aim of the current study was to investigate the tinnitus outcomes 1 year after bilateral cochlear implantation. Secondarily, the tinnitus outcomes in simultaneously and sequentially bilaterally implanted patients were compared. Furthermore, the long-term (3 years) tinnitus outcomes of both study groups were investigated.

## Materials and Methods

### Ethical Considerations

This study was approved by the Human Ethics Committees of all participating centers (NL2466001808), and was registered in the Dutch Trial Register (NTR1722). Written informed consent was obtained from all participants.

### Study Design

Data for the current study were collected as a secondary outcome measure as part of a multicenter randomized controlled trial (RCT). The aim of this RCT was comparing simultaneous bilateral cochlear implantation (simultaneous group) to sequential bilateral cochlear implantation with an inter-implant period of 2 years (sequential group) in adult participants with severe to profound bilateral postlingual SNHL (14, 15).

**Figure 1:**
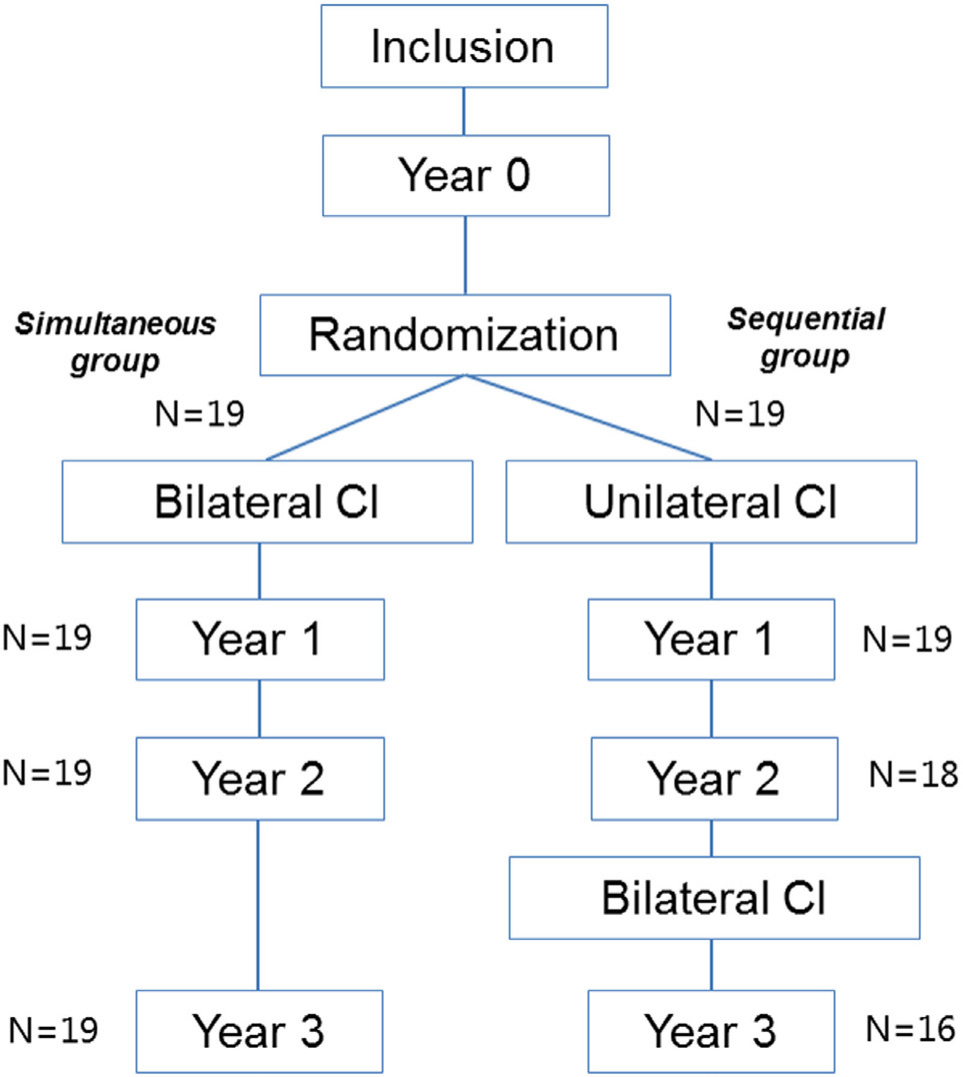
Flowchart of the study design.

Participants were evaluated before implantation and each year after implantation (Figure 1). This study reports the tinnitus outcomes 1 year following bilateral cochlear implantation, which is 1 year after bilateral implantation in the simultaneous group and 3 years after initial implantation in the sequential group. A comparison between the tinnitus outcomes in both study groups 1 year after bilateral cochlear implantation and the long-term tinnitus outcomes (3 years) are also reported.

### Intervention

After giving informed consent, participants were randomly allocated to receive bilateral CIs simultaneously or sequentially with an inter-implant interval of 2 years. All participants were implanted with Advanced Bionics HiRes90K (Advanced Bionics, Sylmar, CA, USA) CIs and were provided with Harmony processors.

### Tinnitus Evaluation

All participants completed the Tinnitus Handicap Inventory (THI) and the Tinnitus Questionnaire (TQ) at each evaluation. Both questionnaires are internationally validated and broadly used.

The THI is a questionnaire regarding tinnitus handicap in daily life. The questionnaire comprises a 12-item functional subscale, an 8-item emotional subscale, and a 5-item catastrophic subscale. The three answer options are “yes,” “sometimes,” and “no,” with scores of 4, 2, and 0, respectively (16). The total score of this questionnaire represents the severity of the tinnitus: slight (0–16), mild (18–36), moderate (38–56), severe (58–76), or catastrophic (78–100) (16, 17).

The TQ consists of 52 questions on emotional and cognitive distress, intrusiveness, auditory perceptual difficulties, sleep disturbance, and somatic complaints. The answer options are “true,” “partly true,” and “not true,” and correspond to scores of 2, 1, and 0, respectively. Forty out of these 52 questions are used to compute the total TQ score (18).

The questionnaires are available in Dutch and a higher score indicates a higher burden of tinnitus.

### Sample Size

Tinnitus burden was a secondary outcome of the original RCT (14, 15). The sample size of this RCT was based on the primary outcome, which was the speech-intelligibility-in-noise. For the current study, the sample size needed to detect statistically significant changes in tinnitus scores after cochlear implantation appeared to be at least 24 participants with preoperative tinnitus. As the presence of tinnitus was not an inclusion criterion of the RCT, the proportion of participants with preoperative tinnitus (*n* = 16) was lower than 24. The chance of detecting a true effect is therefore reduced and therefore we decided to only perform a descriptive analysis of the results.

### Statistical Methods

Tinnitus questionnaire scores were calculated for all participants preoperatively and each year postoperatively. In case of >10% missing data, meaning more than two questions on the THI and more than four questions on the TQ, participants would be excluded from analysis. The THI and TQ were analyzed separately since they have different clinimetric properties and measure tinnitus burden in a different way. Of the THI, the total score was calculated by the sum of all 25 questions. Of the TQ, the total score was calculated by the sum of 40 out of the 52 questions as stated by the manual (18). A participant was considered to have tinnitus when they reached a score higher than zero on either of the questionnaires.

Changes in tinnitus scores were calculated by the subtraction of the preoperative tinnitus score from the postoperative score. Participants were divided into several categories based on their individual tinnitus complaints and evolution over time. Relevant differences in questionnaire scores are called the minimal important changes (MICs) (19, 20). If the score after implantation decreased more than the MIC, a participant was considered to have decreased tinnitus. If the score after implantation increased more than the MIC, a participant was considered to have increased tinnitus. If the difference in score was smaller than the MIC, a participant was considered to have stable tinnitus. If the postoperative score decreased to zero, a participant was considered to have a total suppression of tinnitus. For the THI, the MIC was defined as a difference of seven points between the preoperative and postoperative THI score by the study of Zeman et al. (19). This difference applies for a decrease as well as an increase in score. For the TQ, there are two different MIC scores detected by the study of Adamchic et al. (20). Improvement was defined as a decrease of five points or more in TQ score after implantation. The MIC for deterioration was defined as an increase of one point or more in TQ score after implantation (20).

None of the results were normally distributed, therefore we reported medians and IQRs. All data were analyzed using SPSS for Windows version 21.0.

## Results

### Recruitment and Baseline Characteristics

Between December 2009 and September 2012, 38 participants were included in this study. Nineteen participants were allocated to the simultaneous group and 19 participants to the sequential group (14, 15, 21). Table 1 shows the baseline characteristics.

**Table 1:** Baseline characteristics.

	Simultaneous group	Sequential group
Number of participants	19	19
Male, number (%)	8 (42)	11 (58)
Age at inclusion, years, median (IQR)	52 (36–63)	54 (43–64)
Duration severe hearing loss right ear, years, medians (IQR)	16 (11–25)	17 (9–33)
Duration severe hearing loss left ear, years, median (IQR)	16 (11–25)	18 (9–35)
PTA right ear, decibels, median (IQR)	106 (89–119)	106 (94–111)
PTA left ear, decibels, median (IQR)	108 (89–120)	108 (93–114)
Hearing aid use before CI, number/total	19/19	15/19
Tinnitus prevalence, number/total	9/19	7/19
Preoperative THI score, median (IQR)	22 (8–37)	8 (2–18)
Preoperative TQ score, median (IQR)	20 (12–27)	7 (0–24)

*IQR, inter-quartile range; PTA, pure tone average over 1, 2, and 4 kHz; CI, cochlear implant; THI, Tinnitus Handicap Inventory; TQ, Tinnitus Questionnaire*.

### Loss to Follow-up and Missing Data

In the sequential group, three participants did not receive their second CI due to withdrawal from the study for personal reasons (*n* = 2) and central deafness due to rhesus antagonism that was missed at inclusion (*n* = 1) (Figure 1). Therefore, the 2- and/or 3-year THI and TQ results of these patients were missing. In one other patient, the 3-year TQ results were missing.

## Tinnitus Outcomes 1 Year after Bilateral Cochlear Implantation

### Participants with Preoperative Tinnitus

**Figure 2:**
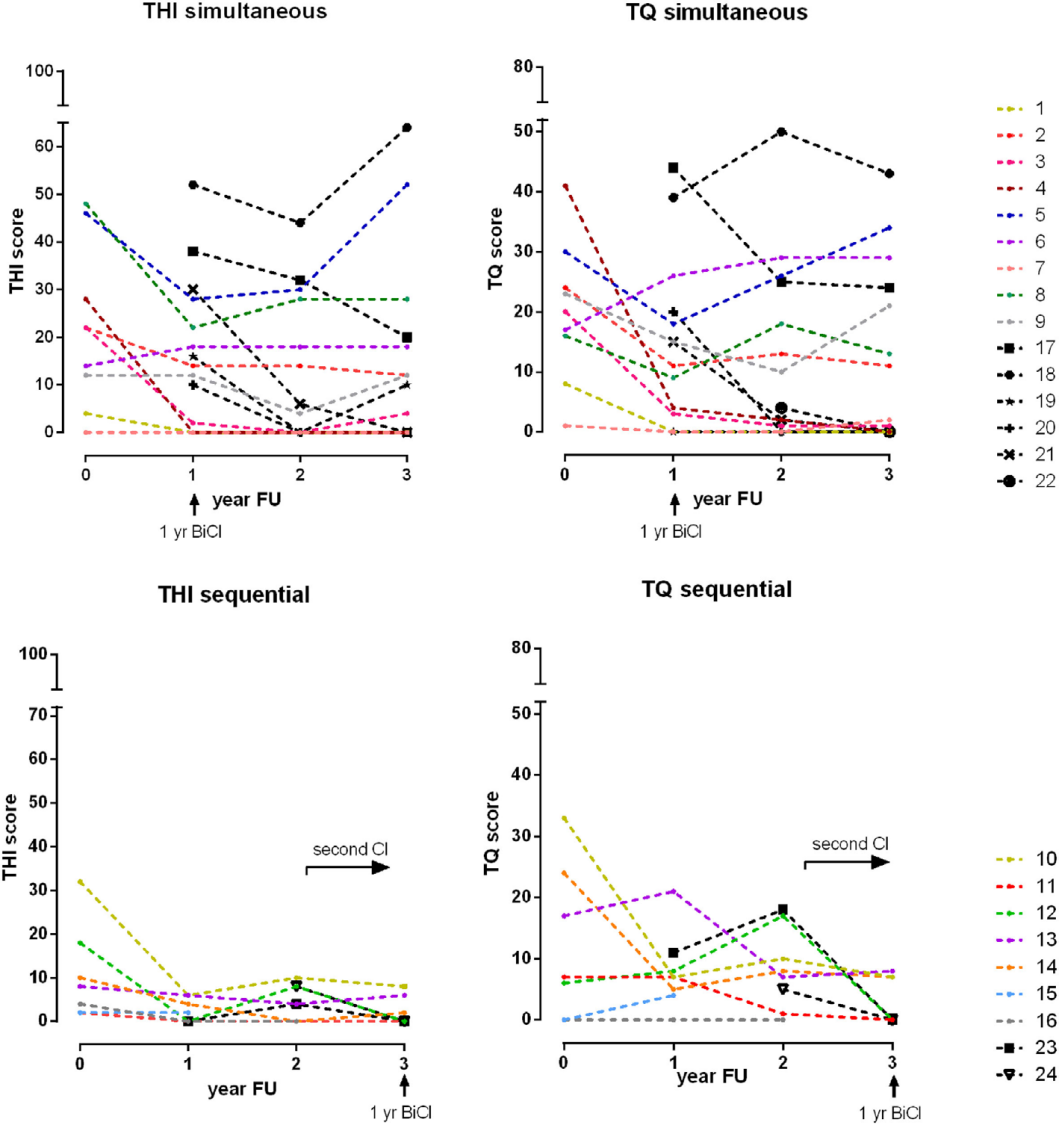
Tinnitus scores and its evolution over time. Legend: the upper graphs show the simultaneous implanted participants and the lower graphs show the sequential implanted participants. The left graphs show the Tinnitus Handicap Inventory (THI) scores and the right graphs show the Tinnitus Questionnaire (TQ) scores. Each colored line represents an individual participant with preoperative tinnitus. Each black line represents an individual participant with induced tinnitus. 1 yr BiCI: 1 year after bilateral cochlear implantation.

Sixteen of 38 participants (42%) experienced tinnitus before cochlear implantation according to the THI or TQ or both, of which 9 patients (47%) in the simultaneous group and 7 patients (37%) in the sequential group. The preoperative and postoperative tinnitus scores of all participants with preoperative tinnitus are shown in Table 2 and Figure 2.

**Table 2:** Tinnitus scores in participants with preoperative tinnitus.

Participant	Group	THI score					TQ score				
		Pre	1-yr	2-yr	3-yr	Δ1-yr BiCI	Pre	1-yr	2-yr	3-yr	Δ1-yr BiCI
1	sim	4	0	0	0	Total ↓	8	0	0	0	Total ↓
2	sim	22	14	14	12	↓	24	11	13	11	↓
3	sim	22	2	0	4	↓	20	3	1	1	↓
4	sim	28	0	0	0	Total ↓	41	4	2	0	↓
5	sim	46	28	30	52	↓	30	18	26	34	↓
6	sim	14	18	18	18	=	17	26	29	29	↑
7	sim	0	0	0	0	No tinnitus	1	0	0	2	Total ↓
8	sim	48	22	28	28	↓	16	9	18	13	↓
9	sim	12	12	4	12	=	23	15	10	21	↓

10	seq	32	6	10	8	↓	33	7	10	7	↓
11	seq	2	0	0	0	Total ↓	7	7	1	0	Total ↓
12	seq	18	0	8	0	Total ↓	6	8	17	0	Total ↓
13	seq	8	6	4	6	=	17	21	7	8	↓
14	seq	10	4	0	2	↓	24	5	8	7	↓
15	seq	2	2	Missing	Missing	Missing	0	4	Missing	Missing	Missing
16	seq	4	0	0	Missing	Missing	0	0	0	Missing	Missing

Median (IQR)	total	13 (4–27)	3 (0–14)	4 (0–14)	5 (0–14)	−8^a^ (−28 to 4)	17 (6–24)	7 (3–14)	8 (1–17)	7 (0–15)	−9^a^ (−17 to −9)
Median (IQR)	sim	22 (8–37)	12 (0–20)	4 (0–23)	12 (0–23)	−8^a^ (−23 to 0)	20 (12–27)	9 (2–17)	10 (1–22)	11 (1–25)	−8^a^ (−15 to −4)
Median (IQR)	seq	8 (2–18)	2 (0–6)	2 (0–9)	2 (0–7)	−8^a^ (−21 to −2)	7 (0–24)	7 (4–8)	7.5 (1–2)	7 (0–1)	−9^a^ (−22 to −7)

THI, Tinnitus Handicap Inventory; TQ, Tinnitus Questionnaire; pre, preoperative; 1-yr, follow-up year 1; 2-yr, follow-up year 2; 3-yr, follow-up year 3; sim, simultaneous group; seq, sequential group; IQR, inter-quartile range; Δ1-yr BiCI, change in tinnitus 1 year after bilateral cochlear implantation (follow-up year 1 simultaneous group, follow-up year 3 sequential group); total ↓, total suppression; ↓, decrease; =, stable; ↑, increase. A score of 0 indicated “no tinnitus.”

*^a^Median difference score = tinnitus score 1 year after bilateral cochlear implantation minus preoperative score*.

The median preoperative THI score was 13 (IQR: 4–27) for all participants with preoperative tinnitus. In the simultaneous group, the median preoperative THI score was 22 (IQR: 8–37) compared with 8 (IQR: 2–18) in the sequential group. The median preoperative TQ score was 17 (IQR: 6–24) for all participants with preoperative tinnitus. In the simultaneous group, the median TQ score was 20 (IQR: 12–27) compared with 7 (IQR: 0–24) in the sequential group (Table 2).

One year after bilateral implantation, the median difference in THI score was −8 (IQR: −28 to 4) and the median difference in TQ score was −9 (IQR: −17 to −9) for all participants with preoperative tinnitus.

One year after bilateral implantation, the tinnitus had totally disappeared in 4 out of 16 participants and decreased in 6 out of 16 participants with preoperative tinnitus according to both the THI and TQ. In four participants, there was disagreement between the THI and TQ (see Table 2). Two of the participants with preoperative tinnitus in the sequential group did not receive a second CI and were therefore unavailable for follow-up.

Although the preoperative and also the postoperative median THI and TQ scores were higher in the simultaneous group, the median difference scores were equal in both groups (Table 2).

### Induction of Tinnitus

As Table 3 and Figure 2 show, 1 year after bilateral cochlear implantation, five participants (participant 17–21) had newly induced tinnitus, all in the simultaneous group. The median THI score was 30 (IQR: 13–45) and TQ score was 20 (IQR: 8–42).

**Table 3:** Tinnitus scores in participants with newly induced tinnitus.

Participant	Group	THI score				TQ score			
		Pre	1-yr	2-yr	3-yr	Pre	1-yr	2-yr	3-yr
17	sim	0	38	32	20	0	44	25	24
18	sim	0	52	44	64	0	39	50	43
19	sim	0	16	0	10	0	0	0	0
20	sim	0	10	0	0	0	20	0	0
21	sim	0	30	6	0	0	15	2	0
22	sim	0	0	8	0	0	0	4	0
23	seq	0	0	4	0	0	11	18	0
24	seq	0	0	8	0	0	0	5	0

THI, Tinnitus Handicap Inventory; TQ, Tinnitus Questionnaire; pre, preoperative; 1-yr, follow-up year 1; 2-yr, follow-up year 2; 3-yr, follow-up year 3; sim, simultaneous group; seq, sequential group. A score of 0 indicated “no tinnitus.”

## Long-Term Results Simultaneous Group

### Participants with Preoperative Tinnitus

The upper part of Figure 2 shows the progression in tinnitus scores in the nine participants with preoperative tinnitus in the 3 years after simultaneous bilateral cochlear implantation. Compared with the preoperative score, 3 years after simultaneous bilateral cochlear implantation the tinnitus had disappeared in two out of nine participants (participants 1 and 4), decreased in two out of nine (participants 2 and 3), and stabilized in one out of nine (participant 9) participants according to both the THI and TQ. In four participants, there was disagreement between the THI and TQ.

### Induction of Tinnitus

As mentioned before, five participants reported a newly induced tinnitus in the first year after implantation. In two of these participants, the tinnitus was reported to be temporary (see Table 2). Two years after simultaneous bilateral implantation, one participant reported newly induced tinnitus, which had disappeared after 3 years.

## Long-Term Results Sequential Group

### Participants with Preoperative Tinnitus

The bottom part of Figure 2 shows the progression in tinnitus scores in the seven participants with preoperative tinnitus in the 2 years after unilateral implantation and in the first year after bilateral implantation. The tinnitus results 1 year after sequential bilateral implantation are described before. Compared with the unilateral situation, the tinnitus had disappeared in two participants (participants 11 and 12). The tinnitus in participant 10 was stable after receiving the second CI. In two participants, there was disagreement between the THI and TQ (participants 13 and 14) and two participants were unavailable for follow-up.

### Induction of Tinnitus

In the 2 years after unilateral implantation, two participants had newly induced tinnitus. The tinnitus disappeared in both participants after sequential bilateral cochlear implantation.

## Discussion

### Key Findings

In this study, we investigated the prevalence and severity of tinnitus before and after simultaneous and sequential bilateral cochlear implantation. We found a relatively low prevalence (42%) and severity of preoperative tinnitus. One year after bilateral implantation, there was a median difference of −8 (IQR: −28 to 4) in THI score and −9 (IQR: −17 to −9) in TQ score in the participants with preoperative tinnitus. Induction of tinnitus occurred in five participants, all in the simultaneous group, in the year after bilateral implantation. Although the preoperative and also the postoperative median THI and TQ scores were higher in the simultaneous group, the median decreases in tinnitus scores were equal after simultaneous and sequential bilateral implantation.

In the simultaneous group, tinnitus scores fluctuated in the 3 years after implantation. Total suppression or decrease of tinnitus burden occurred in four out of nine participants. In the sequential group, low median preoperative tinnitus scores were reported and the tinnitus had disappeared or decreased in four out of seven participants after a 3-year follow-up period. Two participants did not receive a second CI and were therefore unavailable for follow-up. Four participants had an additional benefit of the second CI: a total suppression of tinnitus compared with their unilateral situation.

### Comparison with Literature

The preoperative tinnitus burden we found in our study is lower than described in literature (2). Both the prevalence of tinnitus and the tinnitus burden scores preoperatively were fairly low. On the other hand, the tinnitus induction rate we found was fairly high (2, 5, 6). We presume the low preoperative and high postoperative prevalence of tinnitus is due to a change of the participants’ focus on hearing and all contributing factors. Therefore, it is likely that a participant did not notice the tinnitus before cochlear implantation, but by the increased attention and focus on hearing after implantation the tinnitus appeared and seemed to be newly induced. The onset of new tinnitus could also be independent of the cochlear implantation itself.

Literature on the effect of sequential bilateral cochlear implantation on tinnitus is scarce and the combination of studies that exist showed inconclusive results. Olze et al. (12) found a beneficial effect of the second CI: a further decrease of tinnitus scores in the participants with preoperative tinnitus (*n* = 28). However, Summerfield et al. (11) found a negative effect of the second CI: increase of tinnitus scores in the whole study group (*n* = 24), due to increased tinnitus in 7 of 16 participants with preoperative tinnitus and newly induced tinnitus in 4 of 8 participants without preoperative tinnitus. A possible reason for the discrepancy between these two studies is the difference in tinnitus outcome measurements: Olze et al. used the TQ and Summerfield et al. used a questionnaire concerning tinnitus annoyance (11, 12). In our study, four participants had an additional positive effect of the second CI on their tinnitus burden. The previous studies as well as the current study are studies with a small sample size. Therefore, no firm conclusions can be drawn. To our knowledge, no previous studies on simultaneous bilateral implantation and tinnitus are published.

### Strengths and Limitations

As the design of this study is an RCT, all data were prospectively collected at fixed moments and the same validated questionnaires were used in all participants to measure tinnitus burden. Besides, this is the first study which reports on the effect of simultaneous and sequential bilateral cochlear implantation on tinnitus and therefore this study provides additional evidence to the scarce knowledge.

A limitation of our study is the small sample size, which led to a low statistical power. Therefore, only descriptive analyses of the data were performed. However, it is important to report all outcome measures of an RCT and this study adds knowledge to this field with only two previous studies whose results are contradictive.

Another drawback is the lack of some participant characteristics concerning tinnitus, such as the type of tinnitus, the laterality of tinnitus, and the average and maximum loudness of tinnitus. Also, we did not have information concerning psychological burden of the patients (e.g., anxiety and depression), which is known to affect tinnitus and the overall outcome after cochlear implantation (22). Besides, we assumed the patients suffered from subjective tinnitus; however, we did not specifically evaluate the possibility of somatic modulation, for example. Moreover, we did not have information concerning differences in tinnitus burden with the CI switched “on” and “off” and information concerning the exact time the CI has been worn was also lacking. Another limitation is the difference in preoperative tinnitus severity between the two study groups. This could have resulted in biased postoperative tinnitus outcomes. Tinnitus was a secondary outcome measure in the current study and not the primary complaint of the participants, neither the indication for cochlear implantation. Within the current study, no other tinnitus treatment or personalized medicine was offered to the patients (23).

Furthermore, the measurement of tinnitus is difficult, since it is a subjective symptom and consensus on which questionnaire should be used in a clinical trial is lacking (24). In addition, as holds for the majority of questionnaires, both of the used questionnaires are not validated to measure the effectiveness of an intervention (25). For this reason, the Tinnitus Functional Index was developed in 2012 (which was after the start of the current study (26)). For the THI and TQ, however, the clinically relevant change in scores before and after treatment has been investigated by one study for each questionnaire (19, 20). We used the MIC scores reported in these studies, but it may be questioned whether these MIC scores are representative as for both the THI and TQ, only one study examined these scores. Since there are multiple methods to obtain the MIC and a standard method is lacking, it is plausible that a MIC detected with different methods can vary widely (27). Besides, it is questionable whether these MIC scores are also usable in CI patients as the MIC scores are determined in groups of chronic tinnitus patients. A previous study in chronic tinnitus patients showed a mean THI score of 45 (SD: 23) and TQ score of 40 (SD: 17), both scores are much higher than the preoperative scores we found in the current study (28). This indicates that CI patients with tinnitus may differ from chronic tinnitus patients in terms of tinnitus scores and severity and therefore it is possible that also the MIC scores of the THI and TQ differ in CI patients. The relatively low preoperative tinnitus scores in our study population could also have led to floor effects, which means that it is more difficult to detect improvement.

Future research with larger sample sizes on simultaneous and sequential bilateral cochlear implantation is needed to advance our understanding of the effects of bilateral cochlear implantation on tinnitus. The development or validation of a tinnitus questionnaire to measure treatment effects in CI patients is also needed.

## Conclusion

The present study provides additional evidence to the scarce knowledge on the effect of bilateral cochlear implantation on tinnitus. In general, bilateral cochlear implantation had a positive effect on preoperative tinnitus complaints. The induction of (temporary or permanent) tinnitus was also reported and this should always be taken into account when counseling a patient.

## Ethics Statement

This study was carried out in accordance with the recommendations of the medical ethics committee (MEC) of the Academic Medical Center (AMC) with written informed consent from all subjects. All subjects gave written informed consent in accordance with the Declaration of Helsinki. The protocol was approved by the MEC AMC. This study was also registered in the Dutch Trial Register (NTR1722).

## Author Contributions

All authors have made substantial contributions to the paper. GR collected parts of the data, analyzed the data, and wrote the paper. VK and AZ collected parts of the data and critically revised the paper. YS designed the study, recruited and included participants, collected parts of the data, and critically revised the paper. IS provided advice on statistical analysis and critically revised the paper. RS, RF, JF, and WH recruited and included participants and critically revised the paper. GZ designed the study and critically revised the paper. WG initiated and designed the study, negotiated the funding for the study, and critically revised the paper.

## Conflict of Interest Statement

RF reported receiving nonrestricted grants from Advanced Bionics and being sponsored by a neurotological stipendium from the Heinsius Houbolt Foundation. JF reported receiving nonrestricted grants from Advanced Bionics and MedEl. WG reported receiving nonrestricted grants from Advanced Bionics, MedEl, Oticon, and Cochlear. No other disclosures were reported.
